# Oleanolic Acid and Its Derivatives: Biological Activities and Therapeutic Potential in Chronic Diseases

**DOI:** 10.3390/molecules22111915

**Published:** 2017-11-13

**Authors:** Taiwo Betty Ayeleso, Mashudu Given Matumba, Emmanuel Mukwevho

**Affiliations:** Department of Biochemistry, North West University, Private Bag X2046, Mmabatho 2735, South Africa; taiwo.ayeleso@gmail.com (T.B.A.); mgmatumba@gmail.com (M.G.M.)

**Keywords:** oleanolic acid, diabetes, chromatography, triterpenoid, biological activity

## Abstract

The increasing demand for natural products as an alternative therapy for chronic diseases has encouraged research into the pharmacological importance of bioactive compounds from plants. Recently, there has been a surge of interest in the therapeutic potential of oleanolic acid (OA) in the prevention and management of chronic diseases. Oleanolic acid is a pentacyclic triterpenoid widely found in plants, including fruits and vegetables with different techniques and chromatography platforms being employed in its extraction and isolation. Several studies have demonstrated the potential therapeutic effects of OA on different diseases and their symptoms. Furthermore, oleanolic acid also serves as a framework for the development of novel semi-synthetic triterpenoids that could prove vital in finding therapeutic modalities for various ailments. There are recent advances in the design and synthesis of chemical derivatives of OA to enhance its solubility, bioavailability and potency. Some of these derivatives have also been therapeutic candidates in a number of clinical trials. This review consolidates and expands on recent reports on the biological effects of oleanolic acid from different plant sources and its synthetic derivatives as well as their mechanisms of action in in vitro and in vivo study models. This review suggests that oleanolic acid and its derivatives are important candidates in the search for alternative therapy in the treatment and management of chronic diseases.

## 1. Introduction

Plants have been commonly used as alternative or complementary remedy for chronic diseases, especially in developing countries [1]. The undisputable therapeutic effects of plants have drawn the attention of researchers in the quest for the discovery of bioactive compounds present in plants and its products. Several studies have identified different phytochemicals from plants with biological activities against chronic diseases. Oleanolic acid (OA) is a natural product that has been isolated from several food and medicinal plants [2]. It is a pentacyclic triterpenoid which is abundant in plants of the Oleaceae family such as the olive plant [3,4]. In these plants, OA is often found in the epicuticular waxes where they act as a barrier against pathogens and water loss [5]. Apart from its ecological roles in plants, some pharmacological activities such as anti-oxidant, anti-tumour, anti-inflammatory, anti-diabetic, anti-microbial effects have been attributed to OA in different models of diseases [6,7,8,9,10]. Oleanolic acid has been used as a hepatic drug for over 20 years in China because of its hepatoprotective effect [11]. The exploration of the other biological activities of OA and its synthetic derivatives can lead to the development of potent drugs for the treatment or management of human diseases. Presently, studies on the ability of OA in the amelioration of high fructose-induced oxidative stress and inflammation as well as prevention of metabolic syndrome are on-going in our laboratory. This review consolidates recent reports of biological activities of oleanolic acid and its derivatives. It provides an overview of the current standing of research on the potential of oleanolic acid in the treatment of chronic diseases.

### 1.1. Physical and Chemical Nature of Oleanolic Acid and It Derivatives

**Figure 1:**
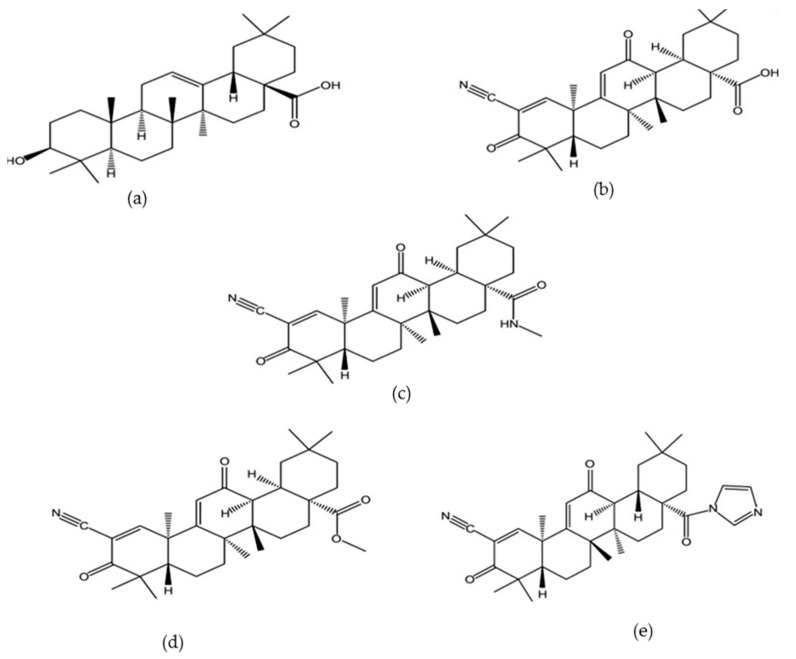
Structures of oleanolic acid and some of its derivatives (**a**) Oleanolic acid ] (**b**) CDDO (**c**) CDDO-Ma ] (**d**) CDDO-Me (**e**) CDDO-Im [17].

Oleanolic acid is a triterpenoid which exists in nature as a free acid or as an aglycone of triterpenoid saponins and it is often ubiquitously found with its isomer, usolic acid [12]. The molecular formula and weight of OA -are C_30_H_48_O_3_ and 456.70 g/mol respectively [13]. It has also served as a framework for additional modifications to achieve semi-synthetic OA derivatives for increased potency, reduced toxicity, increased bioavailability and solubility [11,14,15]. For example, Yu et al. [15] designed two novel oleanolic acid prodrugs by reacting OA with 1,3-cyclopropanyl phosphate ester. The sustained release property of 1,3 cyclopropane esters conferred on the derivatives, improved bioavailability and prolonged shelf life during treatment. Chemical modifications of OA has also led to the development of a number of derivatives (Figure 1) such as 2-cyano-3,12-dioxooleana-1,9(11)-dien-28-oic acid (CDDO) which is over 200,000 times more potent than the parent oleanolic acid [16]. CDDO has also further been chemically modified by addition of methyl groups to obtain CDDO-MA (methyl amide), CDDO-Me (methyl ester) and imidazole CDDO-Im (C28 imidazole) [17]. Some other compounds that have been synthesized from OA include oleanolic acid vinyl boronates [18], esters, oximes and oxadiazole derivatives of oleanolic acid [19].

### 1.2. Occurrences of Oleanolic Acid in Food and Medicinal Plants

Pentacyclic triterpenes including OA are widespread in the fruits, leaves and stem bark of various edible and medicinal plants [20]. Medicinal plants such as *Lantana camara* [21] and *Lisgustrum lucidum* [22] are rich sources of oleanolic acid and they have been used traditionally for the treatment of various diseases. Isolation and characterization of oleanolic acid have been achieved in various plants as shown in Table 1. OA can easily be obtained in high yield from olive plant, its main commercial source [23]. Guinda and colleagues [24] reported that the triterpene contents of the leaves of olive plant are mostly dependent on variety with oleanolic acid being the most abundant triterpenoid in the cultivars investigated. They also reported that geographical origin, stage of development and environmental conditions are other factors that may influence the level of oleanolic acid in plants. Common culinary spices such as garden thyme and clove plants are also sources of oleanolic acid. Apple, loquat, grape, elderberry and sage are some of the fruit plants in which oleanolic acid has also been detected and isolated [20,25].

### 1.3. Extraction, Isolation and Characterization of Oleanolic Acid

Oleanolic acid has been extracted or isolated from different plants using extraction techniques such as soxhlet extraction [26], ultrasound assisted extraction [27] and microwave assisted extraction [28]. Analytical equipment such as thin layer chromatography (TLC) [21], High Performance Liquid Chromatography (HPLC) [22,27] and nuclear magnetic resonance (NMR) [29] are common platforms that have been used in the characterization and identification of OA. Isolation of OA usually involves defatting of the pulverized powder of the plant with a non-polar solvent such as *n*-hexane [30] or petroleum ether [26]. This is typically followed by preparation of the crude extracts, and the isolation of oleanolic acid using techniques such as precipitation and crystallization [21], vacuum liquid chromatography [29] and column chromatography [31]. Like most plant bioactives, a number of parameters are also influential on the extraction and isolation efficiency of OA. Some of these factors include the choice of solvents, concentration of extraction solvents, ratios of solvents to plant material, temperature, duration of extraction and extraction/isolation technique [22,32]. These factors are usually manipulated to optimize the isolation and extraction of OA from its different plant sources.

## 2. Biological Activities of Oleanolic Acid and Its Derivatives

### 2.1. Anti-Tumour/Anti-Cancer Effects of Oleanolic Acid and Its Derivatives

A number of studies have reported the anti-tumour and anti-cancer activities of oleanolic acid against tumour and cancer growth in different in vitro and in vivo models. For example, OA inhibits the growth of transplanted tumour in mice and proliferation of liver hepatocellular cells (HepG2). It was suggested that the anti-tumour activity of OA is through the upregulation of the tumour protein (p53), cyclooxygenase-2 (COX-2) mediated activation of mitochondrial apoptotic pathway and cell cycle arrest [40]. On the other hand, there was an induction of cell death by treatment with a combination of second mitochondrial-derived activator of caspases (SMAC) mimetic BV6 and OA in human hepatocellular cells [41].

SMAC mimetic BV6 is a synthetic selective antagonist of inhibitors of apoptosis (IAP) proteins and hence, also a therapeutic candidate in the treatment of cancer [42]. Furthermore, in osmotic stress-induced breast cancer growth, OA reversed the expression of glycolytic enzymes which were previously enhanced by the hypertonic condition. This reversal efficiently led to a decreased cancer cell proliferation [43]. In human bladder cancer cells, treatment with 50 μM of OA subdued proliferation and enhanced apoptosis of the cells through inhibition of Akt/mTOR/S6K and ERK1/2 (pathways crucial to cell growth, proliferation and survival) signaling [44].

Another specific mechanism of anti-tumour action of OA that has been suggested is the induction of overexpression of miR-122, a protein which has been found to be an important tumour suppressor in some types of cancer [45,46]. OA induced the expression of miR-122 in lung cancer cells up to 9.9 folds following treatment with 60 μg/mL of OA for 8 h [47].

Oleanolic acid methyl ester, a derivative of OA also exhibited cytotoxic effects on human cervical cancer cells (HeLa) through induced apoptosis and reactive oxygen species production in a concentration and time dependent manner [14]. In an attempt to enhance the water solubility of OA, Ren et al. prepared a solid inclusion complex of OA with amino-appended ß-cyclodextrins. Apart from a considerable increase in solubility, they recorded an enhanced in vitro cytotoxicity of the inclusion complex on human cancer cell lines [48]. Generally, these studies suggest that OA and its derivatives can be valuable therapeutic agents against tumour and cancer through their diverse mechanisms of action.

### 2.2. Anti-Diabetic Activity

Diabetes is a complex and progressive disease which results from impaired insulin secretion and/or sensitivity [49,50,51]. It is associated with different metabolic complications which affect body organs such as the eyes, kidney, blood arteries and the nerves [51,52]. Oleanolic acid has been used as therapeutic agent in models of diabetes to improve insulin action, inhibit gluconeogenesis and promote glucose utilization. OA lacks the adipogenic activity unlike the commonly used antidiabetic therapeutics such as insulin or thiazolidinediones that up-regulate glucose transport in periphery and often lead to weight gain [53]. Thus OA can prove to be quite a promising and better therapeutic modality without the adipogenic activity observed with other anti-diabetic or anti-obesity therapeutic agents.

The association between insulin resistance and type 2 diabetes has long been recognized and established [54]. Insulin resistance is the hallmark of type 2 diabetes and a major predictor of its onset [54,55]. In insulin resistant HepG2 cells, treatment with 25 μmol/L of OA improved insulin sensitivity by increasing the expression of insulin receptor substrate 1 (IRS-1) and glucose transporter 4 (GLUT-4) proteins [56]. IRS1 is an important factor in insulin-signaling pathways while GLUT-4 is the major glucose transporter in skeletal muscle, adipose tissues and liver and, hence, both are therapeutic targets in the management of diabetes [57]. In adipose tissue of rats, administration of 25 mg/kg/day of OA supplement for 10 weeks also improved fructose-induced insulin resistance through the IRS-1/phosphatidylinositol 3-kinase/Akt pathway [58].

OA has also demonstrated its ability to inhibit gluconeogenesis and attenuate hepatic insulin resistance. Hepatic insulin resistance in obese condition is considered a major link between type 2 diabetes and non-alcoholic fatty liver disease (NAFLD) [59,60]. Treatments of obese diabetic mice with 20 mg/kg/day for 14 days resulted in reduced body, liver and fat weights, enhanced insulin signaling and inhibited gluconeogenesis [7]. Similarly, one of the findings of our study on oleanolic acid indicates that early postnatal administration of OA is able to mitigate the development of NAFLD in fructose fed adult female rats [61]. This evidence also suggests the hepatoprotective potential of oleanolic acid.

**Figure 2:**
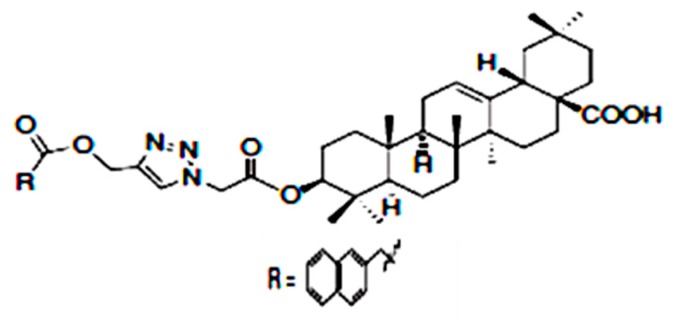
3β-{2-[4-(2-Naphthalen-1-yl)acetoxymethyl-1*H*-1,2,3-triazol-1-yl]acetoxy}olean-12-en-28-oic acid (8 g) [64].

Glycogen phosphorylase is an enzyme which catalyzes the breakdown of glycogen to release glucose into the bloodstream [62]. Its activity contributes to hepatic glucose production and hence, its inhibition is an important approach in the control of hyperglycemia in type 2 diabetes [62,63]. Zhang et al. [64] designed a series of novel derivatives of OA with long alkyl chains or aromatic rings at C3 position in order to enhance its hypoglycemic activity. One of the series synthesized, 3β-{2-[4-(2-naphthalen-1-yl) acetoxymethyl-1*H*-1,2,3-triazol-1-yl]acetoxy}olean-12-en-28-oic acid (Figure 2) showed the strongest activity in the inhibition of glycogen phosphorylase and enhancement of glucose consumption.

Combined therapy is desirable in the treatment of type 2 diabetes to prevent the incidence of secondary failure which sometimes occurs with monotherapy [65,66]. OA has been shown to be able to induce synergistic and complimentary actions with other antidiabetic drugs such as metformin and insulin. Metformin is a widely used drug in the management of type 2 diabetes [67]. In order to appraise the efficacy and synergy of a combination therapy of OA and metformin, the effect of 250 mg/kg of OA combined with 100 mg/kg metformin on db/db mice (a model of diabetes and obesity where the activity of leptin receptor is deficient) was evaluated by Wang et al. [68]. The mice were treated for 4 weeks and compared with monotherapy of each drug. The combined therapy of OA and metformin significantly reduced blood glucose and insulin levels and improved liver pathology when compared with monotherapy treatment in the diabetic mice. Likewise, in type 1 diabetic rat model, 80 mg/kg of OA in synergy with 4IU insulin activated enzymes in the insulin signaling cascade and enhanced insulin-stimulated hypoglycemic activity [69]. These findings suggest that OA may find applications as components in combination therapy for type 2 diabetes.

Inhibition of carbohydrate metabolizing enzymes is an important strategy in the control of postprandial hyperglycemia in type 2 diabetes [70,71]. Castellano et al. [33] isolated OA from the leaves of olive plant and investigated its ability to inhibit α-amylase and α-glucosidase enzymes. The study reported that OA exhibited a potent inhibition of alpha glucosidase. Given the evidences on the antidiabetic potential of OA through multiple and complimentary mechanisms, OA and its derivatives offer promising alternative therapy in the management of diabetes.

### 2.3. Antimicrobial Activity

Oleanolic acid plays a role in defending against pathogens in plants [4]. Hence, OA is expected to possess antimicrobial activity against a wide range of pathogens. In humans, antibiotic therapy is often employed in the treatment of secondary infections sometimes arising from the complications of chronic diseases [72]. Moreover, pathogens have emerged as notable factors, not just in the complications, but also in the progression of chronic diseases [73]. For example, OA exhibited antimicrobial action against *Listeria monocytogenes*, *Enterococcus faecalis* and *Enterococcus faecium* by damaging their cell membranes [74]. To further characterize the function of OA in other microorganisms that causes various ailments, a number of oleanane triterpenoids compounds including oleanolic acid and epi-oleanolic acid were isolated singly from the pericarp of a medicinal food plant, *Akebia trifoliate* [75]. The antibacterial activity of each of these compounds was evaluated against five strains of bacteria. Oleanolic acid showed a moderate activity against *Staphyloccus aureus* and *Bacillus thuringiensis* at 62.5 μg/mL and *Escherichia coli*, *Salmonella enterica* and *Shigella dysenteriae* at 31.2 μg/mL minimum inhibitory concentration (MIC). On the other hand, 3-epioleanolic acid exhibited a stronger antibacterial activity of MIC range (0.9–7.8 μg/mL) which closely compared with the positive reference (kanamycin sulphate) with a MIC range of 1.9–3.9 μg/mL.

Tuberculosis (TB) is a potential chronic disease caused by the bacillus *Mycobacterium tuberculosis* and it is one of the leading causes of death in developing countries [76]. Drug resistance is a major threat to the control and management of TB and one of the strategies employed to overcome drug resistance is the use of combination therapy [77]. Jimenez-Arellanes et al. [78] combined OA with its isomer, ursolic acid (UA) to determine their synergistic antibacterial activity against *Mycobacterium tuberculosis* H37Rv and drug resistant clinical strain (MDR) of tuberculosis in macrophage cell lines and tuberculosis infested BALB/C mice. The study assessed the pulmonary bacilli loads and expression levels of interferon-γ (IFN-γ), tumor necrosis factor-α (TNF-α) and inducible nitric oxide synthase (iNOS). Although OA and UA singly showed antimycobacterial activity, there was also a synergistic intracellular activity of the mixture of both compounds against the tuberculosis strains in the macrophage cell lines. In the infected BALB/C mice, there was a significant reduction in the pulmonary bacilli loads upon treatment with both compounds. Furthermore, there was an increase in expression of iNOS and the cytokines; TNF-α and IFN-γ, which suggests that OA in combination with UA also have immunomodulatory effects that can be harnessed in the control of tuberculosis and possibly other diseases.

There are also reports of OA derivatives that have been designed and evaluated as potential therapeutic agents in the control of microbial diseases. For example, 25 oxime ester derivatives of OA were designed and synthesized by Zhao et al. [11] to study their anti-fungal activities. All the derivatives showed a more potent antifungal activity than the ‘parent’ OA against *Sclerotinia sclerotiorum* and *Rhizoctonia solani* at a concentration of 50 μg/mL. However, they exhibited a much lesser inhibition of glucosamine-6-phosphate synthase (a molecular target of antifungal agents) than the ‘parent’ compound, OA.

### 2.4. Hepatoprotective Ability

The liver is a crucial organ in the metabolic activities of the body, especially drug modification to suit the body. In the modern day, human beings consume lot of drugs per day as such exposing the liver to cytotoxicity. One of the notable bioactivities of OA is the protection of the liver against toxicity and is currently being used as an over the counter hepatic drug in China [11]. In Wistar albino rats, OA from *Flaveria trinervia* was used and had a significant protective effect on ethanol induced liver toxicity by restoring the hepatotoxic serum marker enzymes levels [37]. This study suggested the antioxidant ability of OA as another possible mechanism of its hepato-protective ability. Combination therapy as a means to enhance drug efficacy has been widely used in treatments of various diseases. Gutierrez-Rebolledo et al. [79] evaluated the effect of OA in combined therapy with UA on anti-tubercular drugs induced liver injury. Hepatoxicity has been recognized as a strong side effect of anti-tubercular drug [80,81]. Administration of 100 and 200 μg/mouse/day of OA and UA mixture prevented the steatosis induced by the anti-tubercular drugs, with better effect at treatment with 100 μg/mouse/day.

**Figure 3:**
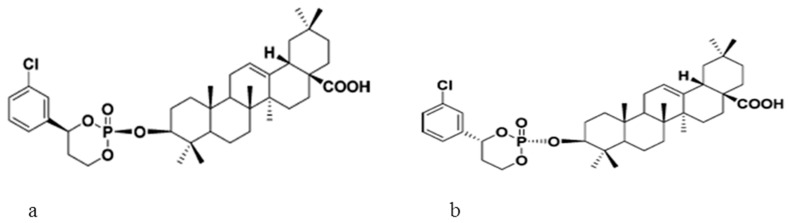
Two novel oleanolic acid prodrugs: (**a**) *cis*-3-*O*-[4-(*R*)-(3-chlorophenyl)-2-oxo-1,3,2-dioxaphosphorinan-2-yl]-oleanolic acid (**b**) *cis*-3-*O*-[4-(*S*)-(3-chlorophenyl)-2-oxo-1,3,2-dioxa-phosphorinan-2-yl]oleanolic acid [15].

In an attempt to change OA metabolism and consequently prolong its shelf life during treatment, Yu et al. [15] developed two novel OA prodrugs (Figure 3). Apart from assessing the metabolism and bio-distribution in rats, the hepato-protective ability of the derivatives was also investigated against carbon tetrachloride (CCl_4_)-induced-liver injury in mice. The increased level of serum hepatic enzymes which were caused by CCl_4_ treatment was reduced significantly upon treatment with the prodrugs. Furthermore, the increased level of malonaldehyde and reduced activities of antioxidant enzymes (GPx and SOD) which are also indicators of hepatic injury were reversed in the mice treated with the synthesized OA derivatives. The study indicated that the prodrugs do not only have improved half-life but also exhibited strong hepatoprotective and antioxidant abilities.

### 2.5. Anti-Hypertensive Effects

Hypertension is a chronic disease which affects many people both in the developing and the developed countries, it is characterized by a chronic increase in systemic arterial pressure above a certain threshold value [82]. Alternative therapy from plants is desirable for the treatment of hypertension as the available synthetic drugs are associated with side effects and secondary failure [83]. Although limited, there are a number of anti-hypertensive reports of OA and its derivatives. For example, the preventive effect of 60 mg/kg OA on glucocorticoid-induced hypertension in rats was evaluated by Bachhav et al. [84]. The use of oleanolic acid significantly prevented an increase in the systolic blood pressure and cardiac lipid peroxidation level. However, there was no significant effect on changes in body and thymus weights which were previously caused by the glucocorticoid treatment. The study proposed a possible involvement of nitric oxide (NO) releasing action of OA in its anti-hypertensive effect. NO is a molecule that has been known to play an important role in the cardiovascular regulatory system [85,86]. In order to further understand the mechanism behind the hypotensive effect of OA and the involvement of NO, Nω-nitro-L-arginine methyl ester (L-NAME)-induced hypertensive rats where NO was completely blocked were treated with OA [87]. OA treatment produced a non-significant increase in the NO*_x_* level. However, this is not enough indication of the involvement of NO in the hypotensive effect of OA and it was suggested that a measure of the level of expression of nitric oxide synthase (NOS) might give a better and major insight into the NO releasing ability of OA. Other parameters such as decreased urine volume, urine sodium and potassium and increased serum creatinine caused by the L-NAME treatment were significantly reversed by OA. The study concluded that the protective effect of OA in L-NAME induced hypertension might be due to diuresis and nephro-protection.

More insights to the hypotensive effect of OA were provided by the study conducted by Madlala et al. [88] on OA and its derivatives; methyl ester OA (Me-OA) and brominated OA (Br-OA). These compounds exhibited vasodilatory actions that were facilitated by both endothelium dependent and independent mechanisms involving COX and vascular muscle K^+^ channels respectively.

### 2.6. Antioxidant Activity

Oxidative stress is known to be involved in the pathogenesis of various chronic diseases and hence antioxidant therapy is a promising strategy for the management and treatment of these diseases [89]. The reports of biological activities of OA have been sometimes attributed to its antioxidant effect. For example, oleanolic acid from *Ligustrum lucidum* was shown to decrease the malonaldehyde (MDA) level and increase superoxide dismutase (SOD) and gluthatione peroxidase (GSH-px) activities in alloxan induced-diabetic rats [90]. The level of MDA (by product of lipid peroxidation), the activities of SOD and GPX (key antioxidant enzymes) are important determinants of antioxidant status in mammalian tissues [91]. Similarly, in an in vitro study, OA increased the production of glutathione and the expression of key antioxidant enzymes [6]. Furthermore, the anti-oxidant property of OA that was isolated from the peel of grape was assessed using ferric reducing antioxidant power (FRAP), 1,1-diphenyl-2-picrylhydrazyl (DPPH) and lipid peroxidation inhibition assays. In this study, the isolated OA exhibited comparable anti-oxidant property with that of commercial anti-oxidant agents [36].

### 2.7. Anti-Inflammatory Potential

Inflammation plays a key role in the development and progression of various diseases such as insulin resistance and diabetes [92], cancer [93] and asthma [94]. In human umbilical vein endothelial cells (HUVECs), OA has been shown to have anti-inflammatory properties by inhibiting the release of liposaccharide (LPS) mediated high mobility group box 1 (HMGB1) and cell adhesion molecules (CAMs) expression [95]. HMGBI is a protein that up-regulates pro-inflammatory cytokines in several inflammatory diseases [96]. Similarly, Lee et al. [97] reported that OA mitigated LPS-induced pro-inflammatory responses by the down-regulation of the expression of nuclear factor-κB (NF-κB) and tumor necrosis factor-α (TNF-α) (biomarkers of inflammation) in vivo and in vitro studies.

Myocarditis is an inflammatory disease of the heart muscle which can progress into chronic heart failure [98]. The anti-inflammatory effect of OA has also been demonstrated in mice with experimental autoimmune myocarditis where it promoted the production of anti-inflammatory cytokines, reduced the production of pro-inflammatory cytokines and ultimately alleviates other symptoms of the disease [99]. Furthermore, the ability of OA to alleviate hepatic insulin resistance in a db/db mouse was partly attributed to its anti-inflammatory activity as evidenced by reduction in the levels of IL-1 β, IL-6, and TNFα in the liver of the mice upon treatment with OA [7].

An oleanolic acid derivative, methyl 3-octanoyloxyiminoolean-12-en-28-oate, also showed anti- inflammatory activity demonstrated by its anti-oedemic effects in rats with carrageenan-induced skin inflammation [100]. These findings suggests that oleanolic acid and its derivatives are promising therapeutic candidate that can be explored in the treatment of inflammation associated diseases.

### 2.8. Anti-Parasitic Activity

The etiological involvement of parasitic infections (especially if left untreated) in chronic diseases has been widely reported [101]. For example, Leishmaniasis, a parasitic infection caused by *Leishmani*a species, can become chronic in the absence of proper treatment [102]. The activity of OA against amastigotes stage of *Leishmania* (L.) *infantum* and *Leishmania* (L.) *amazonensis* was investigated in comparison with maslinic acid (another natural triterpenoid). The study reported that, although maslinic acid showed a better activity against *L. amazonensis*, the oleanolic acid exhibited a relatively higher activity against *L. infantum* with an IC_50_ of 0.999 ± 0.089 μg/mL and selectivity index of 8.111 [103]. Similarly, oleanolic acid exhibited anti-leishmania activity against *L. amazonensis*, *L. braziliensis* and *L. infantum,* agents of three clinical forms of leishmaniasis. Their mechanistic study suggested that OA could interact with sterol 14-α-demethylase (CYP51) (a therapeutic target for leishmaniasis) and deters its oxidant activity [104].

### 2.9. Oleanolic Acid and Its Derivatives in Clinical Trials

A number of OA derivatives have been candidates in phases of clinical trials to determine their safety, dosage, adverse effects and pharmacokinetics profiles. The most popular derivative of OA in clinical trials is bardoxolone methyl (CDDO-Me). CDDO-Me was evaluated in clinical trials, phase 1 for advanced solid tumours and lymphoma in 47 patients by Hong et al. [105]. Bardoxolone methyl was administered orally once daily for 21 days of a 28-day cycle with a starting dose of 5 mg/day. CDDO-Me was well tolerated in the patients with a maximum tolerated dose (MTD) of 900 mg/day. Moreover, it showed anti-tumour activity by activating NRF2 (a transcription factor traditionally deemed as a tumor suppressor) in peripheral blood mononuclear cells (PBMCs) and inhibiting NF-κB and cyclin D1 (a mediator of cell cycle progression) in tumor biopsies. Estimated glomerular filtration rate (eGFR, a measure of renal function) of the patients were also increased and this indicates that CDDO-Me might be able to play a role in the treatment of chronic kidney disease. The most common adverse effects were fatigue, nausea and anorexia which occurred in 40%, 34% and 30% of the patients respectively. However, there was no life-threatening adverse effect associated with CDDO-Me in the trial.

Furthermore, CDDO-Me was evaluated in a phase 2 study for chronic kidney disease (CKD) associated with type 2 diabetes in 227 patients. In the study, treatment with bardoxolone methyl for 52 weeks led to significant improvement in the eGFR among the patients. There were also sustained increases in the eGFR for 4 weeks after the termination of bardoxolone methyl treatment; the study proposed this could be due to a reduction in the inflammation and oxidative stress associated with CKD. The adverse effects among the patients were muscle spasm, mild elevations in alanine aminotransferase levels and gastrointestinal effects which generally ceased without discontinuation of the drug [106]. However, the safety of bardoxolone methyl (CDDO-Me) became questionable when patients treated with the drug in the phase 3 study developed heart related side effects leading to a halt in the trial [107]. Nevertheless, CDDO-Me is currently being assessed in a phase 3 clinical studies to determine its efficacy as a safe therapeutic for connective tissue disease associated pulmonary arterial hypertension [108].

## 3. Conclusions

Oleanolic acid derived from plants as well as its synthetic derivatives has been shown to exhibit different biological activities in various models of diseases through diverse mechanisms of action. This review has highlighted evidences from in vitro and in vivo studies of the ability of OA and its derivatives to reverse or attenuate different diseases and their respective biomarkers. Clinical studies were also reviewed to give an overview of the progress of OA derivatives towards becoming safe and effective therapeutics. It is worthy of note that there are little or no literature evidences of OA itself as a candidate in clinical trials as all clinical studies reviewed were on its synthetic derivatives. This is because, in recent years, major progress has been made in the chemical modification of OA to achieve less toxic, more potent and bioavailable compounds. This is evident from numerous derivatives of OA that are being designed, synthesized and investigated for their biological activities in different in vitro and in vivo studies. There is a clear indication that, OA and its derivatives, if fully explored, have the potential of providing an alternative and cheaper therapy for various chronic diseases.

## Figures and Tables

**Table 1:** Some of the plant sources of oleanolic acid.

Plant Sources	Extraction Methods/Solvents	Isolation Technique/Solvents	Analytical Platforms
*Olea europaea* [33]	Maceration/96% ethanol	Crystallization and filtration	GC-FID *, GC-MS *, DSC *
*Achyranthes aspera* [34]	Continuous shaking extraction, microwave and ultrasonic assisted extraction/methanol	NA	RP-UFLC-DAD *, ATR-FT-IR *
*Aspilia Africana* [35]	Cold maceration/water: methanol (30:70), sequential extraction with water and *n*-butanol	Silica gel column chromatography/CHCl_3_/MeOH (99:1)	TLC
*Monotheca Buxifolia* [29]	Maceration/methanol	Vacuum Liquid Chromatography and Column chromatography/*n*-hexane, chloroform and ethyl acetate	NMR
*Lantana camara* [21]	Defatting with petroleum ether and maceration in ethanol	Precipitation and crystallization/chloroform and methanol respectively	TLC, HPLC, IR *
*Borreria stachydea* [26]	Soxhlet extraction/petroleum ether, Chloroform, ethyl acetate and methanol	Column Chromatography, Thin Layer Chromatography	NMR, GCMS and IR
*Ligustrum lucidum* [22]	Microwave assisted extraction/ethanol, methanol, *n*-butanol and water	N/A	HPLC
*Ligustrum lucidum* [27]	Ultrasound assisted extraction	N/A	HPLC
*Ocimum sanctum*[28]	Microwave assisted extraction/ethanol, methanol and water	N/A	HPLC
*Vitis vinifera* [36]	Sonication/methanol and ethyl acetate	Silica gel Column chromatography/ethyl acetate and *n*-hexane	NMR, EI-MS *
*Flaveria Trinervia* [37]	Soxhlet extraction/chloroform	Thin layer and column chromatography/Hexane and ethyl acetate	IR, ^1^H-NMR
*Syzygium aromaticum* [38]	NS/hexane, dichloromethane, ethyl acetate and methanol	Recrystallization/ethanol	¹H- and ¹³C-NMR
*Satureja Mutica* [31]	Percolation/diethyl ether	Silica gel column chromatography/hexane, chloroform, methanol, ethyl acetate	^1^H-NMR, ^13^C-NMR and MS
*Miconia albicans* [39]	Maceration/*n*-hexane, methylene chloride and ethanol	Vacuum liquid chromatography/*n*-hexane, ethylacetate, ethanol/and High performance liquid chromatography/*n*-hexane, isopropyl alcohol	^1^H-NMR, ^13^C-NMR *

* ATR-FTIR—Attenuated total Reflection-Fourier transform Infrared spectroscopy; DSC—Differential scanning calorimetry; EI-MS—Electron ionization-mass spectroscopy; GC-FID—Gas chromatography-Flame ionization detector; GCMS—Gas chromatography-mass spectroscopy; IR—Infrared spectroscopy; RP-UFLC-DAD—Reverse phase-ultra flow liquid chromatography-diode array detector; MS—mass spectroscopy; NA—not applicable; NS—not specified.
